# Efficacy and safety of the enzymatic mixture - Lipase, collagenase and hyaluronidase - In the treatment of moderate to severe submental fat: A prospective cohort study

**DOI:** 10.1016/j.heliyon.2024.e25759

**Published:** 2024-02-10

**Authors:** Rita Jabbour, Fadi Farah, Farid Mallat, Eddy Saad, Karl Semaan, Roger Haber, Josiane Helou

**Affiliations:** a Dermatology Department, Hôtel-Dieu de France Hospital, Beirut, Lebanon; b Radiology Department, Hôtel-Dieu de France Hospital, Beirut, Lebanon; c Faculty of Medicine, Saint Joseph University, Beirut, Lebanon; d Internal Medicine Department, Hôtel-Dieu de France Hospital, Beirut, Lebanon; e Dermatology Department, University of Illinois, Chicago, USA

**Keywords:** Submental fat, Lipase, Collagenase, Hyaluronidase

## Abstract

**Purpose:**

To study the effect of the enzymatic mixture: Lipase, Collagenase and Hyaluronidase in the treatment of submental fat.

**Methods:**

A monocentric prospective cohort study including 10 female patients, aged between 18 and 65 years old, who received treatment for submental fat with a mixture of Lipase, Collagenase, and Hyaluronidase. The treatment protocol consisted of one treatment session every 21 days for a total of 3 sessions. In each session, 4 ml of the enzymatic mixture (1 ml of Collagenase GH PB20, 1 ml of Hyaluronidase PB 3000 and 2 ml of Lipase PB 500) + 2 ml of Lidocaine 2% were injected in the submental fat (SMF). Efficacy was assessed four weeks after the last session. Co-Primary Outcome was defined as the improvement of ≥ 1-point in Clinician-Reported and Patient-Reported Sub-mental Fat Rating Scales (CR-SMFRS and PR-SMFRS). Secondary Outcomes included score reductions in Patient-Reported Sub-mental Fat Impact Scale (PR-SMFIS), ≥10% reduction in submental fat pad thickness by ultrasound, and Subject Self-Rating Scale (SSRS) responses of 4, 5, or 6.

**Results:**

The Co-Primary outcome was achieved in 9 out of 10 patients. A considerable reduction of 22.8% in the PR-SMFIS was observed. Furthermore, 9 out of 10 patients expressed overall satisfaction with the treatment. Submental fat reduction of more than 10% was observed in 9 out of 10 patients in neutral position and in all patients in flexed position. Adverse effects were only limited to local reactions.

**Conclusion:**

The enzymatic mixture of Lipase, Collagenase and Hyaluronidase is an effective and safe minimally-invasive method for the reduction of SMF that can be used alone or in conjunction with other treatment modalities.

## 1 Introduction

Excess submental fat (SMF) is a common cosmetic concern due to accumulation of fat in the preplatysmal compartment of the neck [1]. As a result, the mandibular line definition is lost, which impacts patient's confidence and self-esteem [2]. Surgical liposuction has been the primary method for treating SMF [3]. Although liposuction may provide reliable results, many patients are reluctant to undergo surgery due to associated ecchymosis, skin laxity, risk of neurological and vascular complications, financial burden and surgical recovery time [4]. With patients and physicians seeking less invasive methods, the use of non-surgical fat reduction methods is gaining popularity. These new techniques include submental cryolipolysis, percutaneous radiofrequency, laser techniques, high-intensity focused ultrasound and injectable chemical lipolysis [5]. In March 2015, the injectable deoxycholic acid formulation, ATX-101, received the FDA approval for the reduction of SMF [6]. It causes preferential fat cell membrane disruption, thus inducing lipolysis [7]. Since then, this procedure has remained the only approved injectable treatment for this indication. Recently, recombinant enzymes with lipolytic properties have also been used in this field. These enzymes are produced by recombinant proteins and can be obtained rapidly, in large quantities, and with high purity [8]. However, there are scarce data in the literature on the efficacy and safety of these enzymes in treating SMF.

A mixture of three enzymes is being marketed and used in several countries around the world to treat fat accumulation: Lipase PB500 (from *Thermus thermophilus*), Collagenase GHP220 (from *Clostridium histolyticum*), and Hyaluronidase PB3000 (from *Streptococcus pyogenes)* (LCH) *By* PROTEOS BIOTECH SL. However, no studies have been conducted to evaluate the efficacy and safety of this enzymatic mixture. This monocentric prospective cohort study aims to assess the efficacy and safety of this in the treatment of SMF.

## 2 Methods

### 2.1 Study method

This is a single-center, prospective cohort observational study. Patients who presented to our dermatology department between May 2022 and October 2022 to treat their SMF with LCH enzymatic mixture were screened. Our study aimed to assess both their satisfaction and clinical improvement as well as potential adverse events following the treatment.

### 2.2 Patient selection

Eligible subjects were female patients aged between 18 and 65 years old having moderate to severe submental fat for at least 6 months and a stable weight for the past 6 months.

Patients were excluded if they had a BMI >40 kg/m2, severe skin laxity, a history of allergy to one of the components of the enzyme mixture, previous treatment with deoxycholic acid, enzyme mixtures, radiofrequency, laser, chemical peeling, botulinum toxin, previous liposuction of the neck and neck surgery in the last 6 months preceding the intervention. Were also excluded patients with previous chin, mandibular or cervical filler in the last 12 months prior to enrollment.

### 2.3 Injection technique

Anatomical landmarks were identified in each patient to minimize the risk of any complications related to the adjacent neurovascular and glandular neck structures. Injections were avoided above a line drawn 1.0–1.5 cm below the inferior border of the mandible to prevent any damage to the marginal mandibular nerve potentially leading to paresis. Other important anatomic landmarks included the anterior border of the sternocleidomastoid muscle and the thyroid cartilage inferiorly. Once the markings were defined, an injection grid of 1 cm spacing between injection sites was drawn. Patients received 0.2 mL aliquots per injection site delivered through a 30-gauge needle.

Each patient received 1 treatment session every 21 days for a total of 3 sessions. In each session, 4 mL of the enzymatic mixture (1 mL of Collagenase GH PB220, 1 mL of Hyaluronidase PB 3000, 2 mL of Lipase PB 500) + 2 mL of Lidocaine 2% were injected into the SMF.

**Fig. 1:**
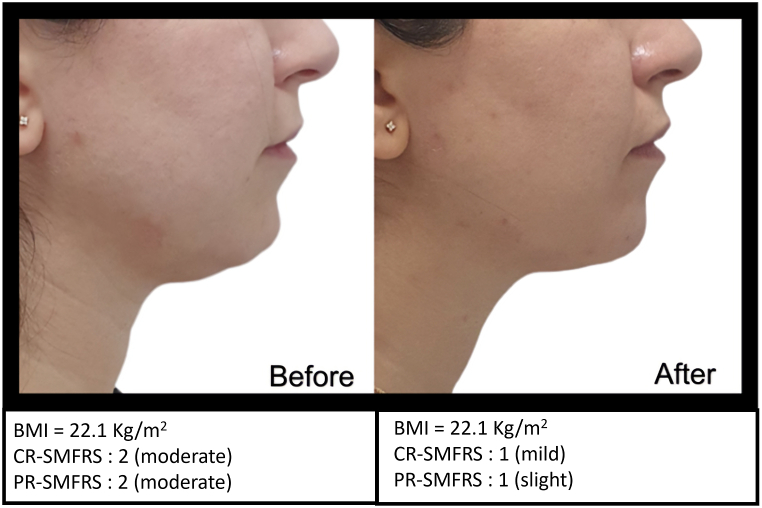
Profile view of patient at screening and 15 days after the last session.

**Fig. 2:**
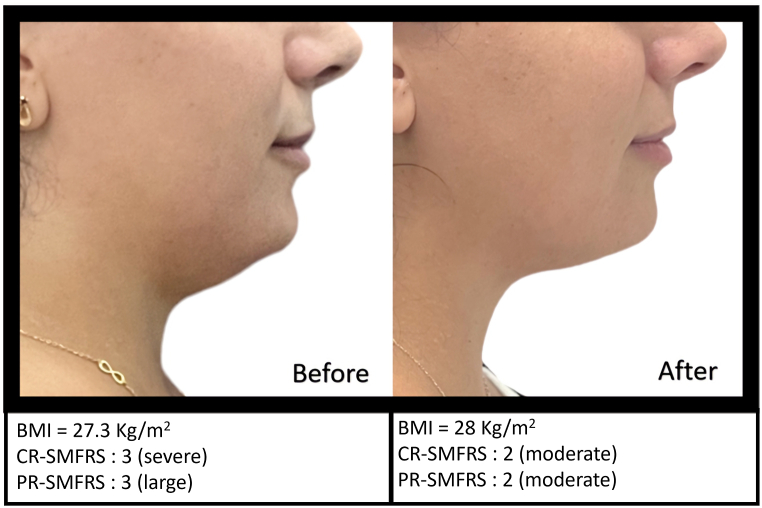
Profile view of patient at screening and 15 days after the last session.

**Fig. 3:**
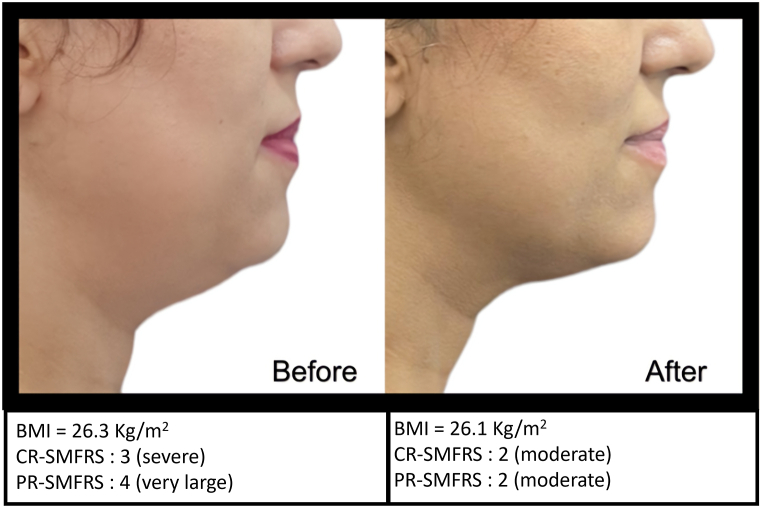
Profile view of patient at screening and 15 days after the last session.

Because this product is composed of recombinant enzymes with high specificity and substrate selectivity, it acts quickly by digesting the targeted constituents of the soft tissue. For this reason, standardized photographs of the profile views were obtained for documentation of treatment effect at screening and 15 days after the last session. (Fig. 1, Fig. 2, Fig. 3). For standardized positioning, the camera's height was adjusted to align with the Frankfort plane, an imaginary line that passes through the top of the ear canal and the lower margin of the eye socket. The distance between the camera and the top of the ear canal was 30 cm. Patients were weighed at baseline and 15 days after the last session.

A soft tissue ultrasound of the preplatysmal fat was performed at the neutral position and after neck flexion to measure the thickest fat pad at screening and 15 days after the last session. All the ultrasounds were performed by the same operator to reduce operational bias.

Four scores were used: Clinician-Reported and Patient-Reported Sub-mental Fat Rating Scales [CR-SMFRS and PR-SMFRS], PR-SMFIS*: Patient-Reported Sub-mental Fat Impact Scale and SSRS: Subject Self-Rating Scale (Appendix).

In each visit, screening for potential adverse events was conducted.

The hospital Ethics Committee reviewed and approved the protocol. All subjects enrolled in the study provided signed informed consent.

### 2.4 Evaluation and trial outcomes

Efficacy was assessed 4 weeks after the last session.

Co-Primary Outcome was defined as the improvement of more than 1 point in the CR-SMFRS and PR-SMFRS.

Secondary Outcomes were score reduction compared to baseline of the PR-SMFIS, reduction of ≥ 10% in the submental fat pad as measured by ultrasound in both neutral and flexed positions, and an SSRS response of 4, 5 or 6.

### 2.5 Statistical analysis

Mean and standard deviation were computed for each quantitative variable and proportions were also calculated for each qualitative variable.

The values for the following parameters: CR-SMFRS, PR-SMFRS, sub mental fat thickness in both flexion and neutral positions and the score for each individual question as well as the average score on the 6 questions were compared before and after the injection using a Wilcoxon matched pairs signed-rank test. The significant threshold was set at 0.05. All calculations were done using R (v 4.2.2) on Rstudio (v 2022.7.2.576).

## 3 Results

### 3.1 Baseline characteristics

10 female patients were included in this study. Baseline characteristics is described in Table 2. Mean age was (mean [SD]) 41.8 (14.8), mean BMI was 26.34 (2.6) (Table 1).

**Table 1:** Patients' characteristics at baseline (n = 10). SD: standard deviation; CR-SMFRS: Clinician-Reported Sub-mental Fat Rating Scale; PR-SMFRS: Patient-Reported Sub-mental Fat Rating Scale.

Baseline characteristics	
Age, mean (SD), years	41.8 (14.8)
Weight, mean (SD), kg	70.1 (9.2)
Height, mean (SD), cm	163 (5.7)
Fitzpatrick skin type, n	
II	2
III	8
CR-SMFRS, mean (SD)	2.6 (0.5)
CR-SMFRS, n	
Grade 2	4
Grade 3	6
PR-SMFRS, mean (SD)	2.8 (0.8)
PR-SMFRS, n	
Grade 2	4
Grade 3	4
Grade 4	2

**Table 2:** Ultrasound measured submental fat (SMF) pad thickness (NP:neutral position, FP: flexed position).

	SMF thickness before in NP (cm)	SMF thickness after in NP (cm)	Percentage of reduction	SMF thickness before in FP (cm)	SMF thickness after in FP (cm)	Percentage of reduction
Patient 1	1.31	0.82	37.4	1.92	1.22	36.5
Patient 2	0.82	0.8	2.4	1.02	0.86	15.7
Patient 3	1.2	0.7	41.7	1.39	0.94	32.4
Patient 4	1.36	0.65	52.2	1.52	0.9	40.8
Patient 5	1.53	0.92	39.9	1.7	1.29	24.1
Patient 6	1.22	0.8	34.4	1.33	0.99	25.6
Patient 7	1.1	0.79	28.2	1.4	1.12	20.0
Patient 8	1.2	0.96	20.0	1.35	1.1	18.5
Patient 9	1.9	1.6	15.8	2.5	1.8	28.0
Patient 10	1.4	1.1	21.4	1.65	1.4	15.2
**Mean Value**	**1.3**	**0.91**	29.34*	**1.57**	**1.16**	25.66*

^a^p-value <0.01.

### 3.2 Efficacy

Co-Primary outcome was reached in 9 out of 10 patients with an amelioration of ≥ 1 grade in CR-SMFRS and PR-SMFRS, 4 weeks after the last injection session.

**Fig. 4:**
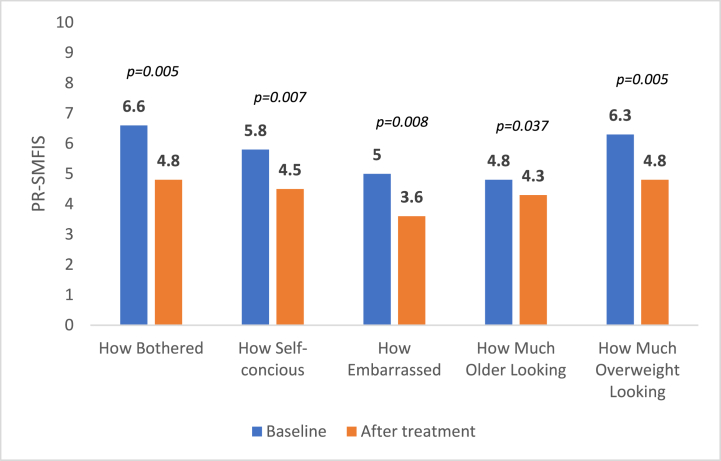
PR-SMFIS at baseline and 4 weeks after the last treatment session.

Patients had a higher psychological impact from the SMF at baseline, with a mean (SD) PR-SMFIS Total Scale Score of 5.7 (0.89) with 9 patients having a score ≥ 5. At week 4 after the last treatment session, PR-SMFIS dropped significantly to 4.4 (0.67) (P < 0.005), a total reduction of 22.8%. Similar results were seen with the individual component scores (Fig. 4).

Submental fat pad measurement by ultrasound was performed in all patients. The mean (SD) fat pad thickness in neutral and flexed position of the neck was 1.3 cm (0.28) and 1.58 cm (0.41) at baseline, versus 0.91 cm (0.27) and 1.16 cm (0.28) 2 weeks after the last injection, respectively (*p =* 0.005 and *p* = 0.006, respectively).

A reduction of more than 10% of the submental fat pad thickness was observed in 9 out of 10 patients in neutral position and in all patients in flexed position (Table 2).

Concerning the SRSS response 4 weeks after the last treatment session, 2 patients were extremely satisfied (SRSS 6), 5 patients were satisfied (SRSS 5), and 2 patients were slightly satisfied (SRSS 4). One patient was neither satisfied nor dissatisfied (SRSS 3) and was considered a non-responder.

### 3.3 Safety

All adverse events (AEs) were local reactions (Table 3). Most AEs were transient and resolved within the treatment interval without sequelae. The most common AE is swelling, described in 4 patients. It occurred directly after the injection and resolved within 48 hours. One patient developed cellulitis at the site of injection 2 days after her third session. This patient went to the beach 3 hours after the procedure, despite our recommendation to avoid saunas, baths, jacuzzis or swimming on the day of the treatment. She was successfully treated with Amoxicillin/Clavulanate 1g twice daily for 7 days. No marginal mandibular nerve paresis nor dysphagia were reported. No serious adverse nor allergic events were reported.

**Table 3:** Adverse events reported following injections of the enzymatic mixture.

Adverse events	n = 10
Injection site reaction	
Swelling	4
Ecchymosis	3
Discomfort	2
Erythema	1
Induration	1
Pain	1
Cellulitis	1
Pruritus	0
Nodule	0
Paresthesia	0
Hematoma	0
Ulceration	0
**Non injection site events (headache, nasopharyngitis, dysphagia)**	0

## 4 Discussion

To our knowledge, this is the first study to assess the efficacy and safety of the enzymatic mixture of lipase, collagenase and hyaluronidase in the treatment of SMF. In this study, LCH was associated with significant improvement in SMF, percieved both by clinicians and patients. Subjective clinical improvement is reflected by an increase of ≥ 1 grade in CR-SMFRS and in PR-SMFRS and an improvement in the SRSS response, and was seen in 9 out of 10 patients (patient 2 was a non-responder). The ultrasound measurement of the thickness supported these results, with 9 patients showing a reduction of more than 10% in the SMF thickness in neutral and flexed positions. Patient 2 had only a 2% reduction in SMF thickness in neutral position, and a 15.6% reduction in SMF thickness in flexed position. This means that both the subjective and objective means of evaluations were concordant.

There is a significant impact of the submental region on self-perception and overall facial appearance [2]. In our study, patients had a psychological impact from their SMF at baseline defined by a mean PR-SMFIS of 5.7. The highest PR-SMFIS scores were given by the patients to the degree of bothersome caused by the SMF (mean response = 6.6), and how much overweight they look due to their SMF (mean response = 6.3). These 2 scores dropped significantly 2 weeks after the last treatment with the highest degree of reduction compared to other questions in the PR-SMFIS score. This means that the degree of bothersome and the self-perception of overweight are highly affected by SMF and are highly responsive to treatment.

To compare the results of our study with those of the REFINE-1 phase 3 trial involving ATX-101 for SMF, it's worth noting that in our study, 90% of participants achieved the primary endpoint, which required a ≥ 1-grade improvement in both CR-SMFRS and PR-SMFRS. In the REFINE-1 trial, this primary endpoint was achieved by 70% of participants. In terms of the reduction in PR-SMFIS scores, our study showed a 22.8% reduction, while ATX-101 in the REFINE-1 trial demonstrated a more substantial 51% reduction. It's important to acknowledge that our study and the REFINE-1 trial used different imaging modalities for evaluating SMF volume. Our study employed ultrasound to measure the fat pad in the submental region, while the REFINE-1 study used magnetic resonance imaging (MRI). Notably, in our study, 90% of participants were responders on the US measurement of SMF volume reduction, compared to 46.3% in the REFINE-1 trial on the MRI measurement of SMF volume reduction [9].

All AEs were limited to the injection sites, with swelling and bruising being the most common AEs. Swelling developed directly after the injection, mostly due to the volume of the enzymatic mixture injected and resolved within 48h. No serious AEs or systemic allergic reaction were reported. Marginal mandibular nerve paresis, that presented as an asymmetric smile, has been reported in the phase III ATX-101 trials (REFINE-1 study), where marginal mandibular nerve paresis was reported in 4.3% of treated subjects [9]. This led to the recommendation that injections should not be given above a line drawn 1.0–1.5 cm below the lower edge of the mandible, from gonion to mentum, to reduce the risk of injury. In our study no cases of marginal mandibular nerve paresis were reported. Moreover, dysphagia was reported in 1.6% of ATX-101 treated subjects in REFINE-1 study [9], but none was reported in our study. One case of cellulitis occurred in our study and resolved with antibiotics without complications.

Four weeks after the last treatment, when patients presented for evaluation of efficacy, no adverse effects other than those previously mentioned were reported. Follow-up at 3 and 6 months is recommended to assess the occurrence of skin sagging following the procedure and/or recurrence. This could also determine any potential need for treatment repetition at regular intervals.

This is the first study that assesses the effectiveness of the LCH enzyme mixture in SMF. Each enzyme in this mixture plays an important role in adipolysis process. Lipase causes the hydrolysis of triglycerides accumulated in adipocytes, transforming them into monoglycerides and free fatty acids. These lipolysis products have a greater diffusing capacity, thus their elimination through lymphatic drainage is much more efficient [8].

In a study conducted by Chen et al., subcutaneous injection of collagenase rColH (E451D) in minipigs induced a decrease in the thickness of adipose tissue, identified by ultrasound measurements [10]. In fact, adipocytes are attached to the extracellular matrix (ECM) via collagen in adipose tissues. Degradation of ECM by collagenase induces the apoptosis of adipocytes. This was confirmed by the histopathological study of the adipose tissue that showed marked destruction of adipose tissue and fibrosis. This is regarded as evidence of adipocyte apoptosis following subcutaneous collagenase injection [10].

Dokoshi et al. demonstrated that hyaluronic acid (HA) accumulated during maturation of adipocytes in vitro and was associated with increased expression of preadipocyte factor 1, zinc finger protein 423, and early B cell factor 1. HA is necessary for adipocyte maturation, and digestion of HA by administration of soluble hyaluronidase or transgenic expression of hyaluronidase 1 was shown to inhibit adipogenesis in vitro and in vivo [11].Beyond injectable lipolytic agents, several other effective non surgical techniques for fat reduction are prevalent in cosmetic procedures. Cryolipolysis freezes and eliminates fat cells by controlled cooling, gradually reducing localized fat deposits [3]. Radiofrequency treatments employ thermal energy to target fat cells, stimulating collagen production and tightening the skin [12]. Ultrasound Therapy and High-Intensity Focused Ultrasound (HIFU) disrupts fat cells and tightens the skin through targeted ultrasound energy [13].

However, enhancing the definition of the lower face doesn't necessarily require fat dissolving techniques. Alternatively, fat grafting, is a procedure that involves the transfer of a patient's own harvested fat cells obtained by liposuction from donor sites such as the abdomen, the thighs, the buttocks, or even the SMF. This technique offers a method for volumetric enhancement and contouring. By strategically placing these fat cells in targeted areas, such as the jawline and chin, fat grafting serves to refine contours and diminish the appearance of a double chin or submental fullness [14].Fat grafting offers the advantage of using the patient's own tissue, reducing the risk of allergic reactions or rejection [15]. This meticulous approach allows for precise sculpting and customization, tailoring the procedure to align with the patient's specific aesthetic goals.

This study has a few limitations. First, it is a prospective observational monocentric cohort study. A randomized double blinded controlled clinical trial will be needed to confirm our findings. The second limitation is the small number of included patients. Third, the evaluation was conducted 4 weeks after the third session in all patients, regardless of the initial size of the SMF. Compared to patients with milder SMF, those with larger SMF volume might however need more sessions to achieve clinical improvement. Longer periods of observations may be required to capture the full extent of the clinical response. Moreover, longer term data are also required to confirm the safety findings from this analysis and evaluate the incidence and extent of recurrence.

In conclusion, the injectable mixture of LCH appears to be a promising minimally-invasive method for the reduction of SMF that can be used alone or in conjunction with other treatment modalities to enhance the definition of the lower face. More robust studies are needed to confirm the safety and efficacy of this enzymatic mixture.

## Data Availability statement

Data associated with this study has not been deposited into a publicly available repository. All data are included in article and/or supplementary material and are referenced in this article.

## Ethics statement

This study has been approved by the Saint Joseph University Ethics Committee (Code: CEHDF 2027).

All participants provided informed consent to participate in the study and for the publication of their anonymised case details and images.

## CRediT authorship contribution statement

**Rita Jabbour:** Writing – original draft, Visualization, Software, Resources, Data curation. **Fadi Farah:** Writing – original draft, Visualization, Software, Resources, Data curation. **Farid Mallat:** Writing – original draft, Visualization, Formal analysis, Data curation. **Eddy Saad:** Visualization, Software, Formal analysis, Data curation. **Karl Semaan:** Visualization, Software, Formal analysis, Data curation. **Roger Haber:** Writing – review & editing, Validation, Methodology, Conceptualization. **Josiane Helou:** Writing – review & editing, Validation, Supervision, Project administration, Methodology, Investigation, Data curation, Conceptualization.

## Declaration of competing interest

The authors declare that they have no known competing financial interests or personal relationships that could have appeared to influence the work reported in this paper.
