# Supplementary material for: Efficacy and safety of the enzymatic mixture - Lipase, collagenase and hyaluronidase - In the treatment of moderate to severe submental fat: A prospective cohort study
Source: Heliyon. 2024 Feb 10;10(4):e25759. doi: 10.1016/j.heliyon.2024.e25759 (PMC10875420; doi:10.1016/j.heliyon.2024.e25759)
Supplement: Multimedia component 1 [file mmc1.docx]

**CR-SMFRS Submental convexity evaluated by clinician on a 5-point ordinal scale (0–4) with:**

0 = absent

1 = mild

2 = moderate

3 = severe

4 = extreme

**PR-SMFRS SMF evaluated by subject on a 5-point ordinal scale (0–4) with:**

0 = no chin fat at all

1 = a slight amount of chin fat

2 = a moderate amount of chin fat

3 = a large amount of chin fat

4 = a very large amount of chin fat

**PR-SMFIS Psychological impact of SMF on self-perception of 5 emotional and visual characteristics related to the appearance of submental fullness assessed with the following items:**

1. How bothered are you by the appearance of your chin fat?
2. How self-conscious are you about the appearance of your chin fat?
3. How embarrassed are you about the appearance of your chin fat?
4. How much older do you look because of your chin fat?
5. How much overweight do you look because of your chin fat?

Each item rated on an 11-point numeric scale (0–10)

Scores for the 5 items were combined to generate a PR-SMFIS Total Scale Score

Lower scores indicate improvement or reduced negative impact of these items

**SSRS Overall satisfaction with the appearance of face and chin evaluated by subject on a 7-point scale (0–6) with:**

0 = extremely dissatisfied

1 = dissatisfied

2 = slightly dissatisfied

3 = neither satisfied nor dissatisfied

4 = slightly satisfied

5 = satisfied

6 = extremely satisfied

A responder was a subject whose response was 4, 5, or 6
